# Single oral dose cure of lethal *P. yoelii* with a new iron chelator in human clinical trials for iron overload

**DOI:** 10.1186/1475-2875-9-S2-P54

**Published:** 2010-10-20

**Authors:** Abhai Tripathi, Hugh Rienhoff, David Sullivan

**Affiliations:** 1 The Malaria Research Institute, W. Harry Feinstone Department of Molecular Microbiology and Immunology,The Johns Hopkins Bloomberg School of Public Health, Baltimore, MD, USA; 2 FerroKin BioSciences, Inc. San Carlos, CA, USA

## Background

Iron chelators for the treatment of malaria have proven therapeutic activity *in vitro* and *in vivo* in both humans and mice, but their clinical use has been limited by the unsuitable adsorption and pharmacokinetic properties of the few available chelators. FBS0701 is an oral iron chelator currently in Phase 2 human studies for the treatment of transfusional iron overload. The drug has very favorable absorption and PK properties allowing for once-daily use to deplete circulating free iron. Once-daily administration of FBS0701 in humans at well tolerated doses can achieve plasma concentrations in the high μM range.

## Materials and methods

*P. falciparum* drug inhibition was performed with Sybrgreen drug inhibition assays. Murine malaria was initiated by intraperitoneal inoculum of Balb/c mice followed for 30 days.

## Results

**Figure 1:**
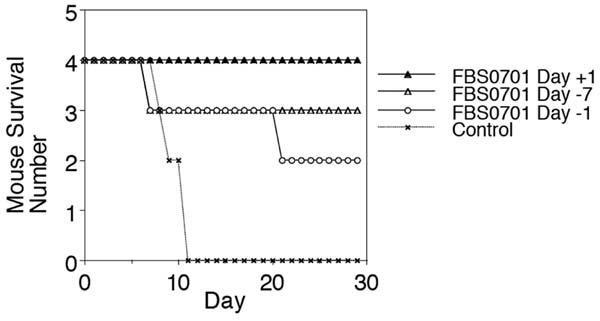
Four mice were inoculated with 10 million lethal *P.yoelii* parasites. The survival curve shows complete protection with Day +1 dosing and significant protection in animals pre-treated Day -7 and Day -1.

FBS0701 has potent IC_50_ of 5 μM in contrast to the IC_50_ for deferiprone and deferoxamine at 15 and 30 μM respectively. The FBS0701 iron chelator was not antagonistic to the quinoloines or artemisinin in *P. falciparum in vitro* tests. In the *P. berghei* Thompson suppression test, 100 mg/kg reduced parasitemia at day three and prolonged survival, but did not cure mice. The number of daily doses - one, three or seven - did not significantly alter outcome in *P. berghei* infection. We then pre-treated mice with a single oral dose of 100 mg/kg of FBS0701 seven days or one day before infection with lethal *P. yoelii* 17XL. We also treated with a single dose one day after *yoelii* infection. All mice treated with a single dose one day following infection survived to 30 days in Figure 1. All untreated mice died by day 11. All FBS0701-treated mice had reduced parasitemia on day three. On day 16, the surviving FBS0701-treated mice had a significant reticulocytosis with more than 70% reticulocytes. Only 10 to 20% of mature erythrocytes were infected on day 16 in contrast to 40-60% of reticulocytes. By Day 23 post-infection, reticulocytosis and parasitemia had resolved.

## Conclusion

FBS0701 demonstrates a single oral dose cure of the lethal *P. yoelii* model. Significantly, this effect persists after the chelator has cleared from plasma. FBS0701 may find clinically utility as monotherapy, a malarial prophylactic or, more likely, in combination with other antimalarials.

